# Trace Elements and Depressive Symptoms in Coronary Artery Disease: A Systematic Review of Sparse and Predominantly Indirect Evidence

**DOI:** 10.3390/ijms27093805

**Published:** 2026-04-24

**Authors:** Jakub Marek Baran, Zuzanna Waszak, Joanna Jarzębska, Damian Grusiecki, Maja Śmigielska, Wacław Kochman, Ewelina A. Dziedzic

**Affiliations:** 1 Department of Cardiology, Bielanski Hospital, 01-809 Warsaw, Poland; jak.baran00@gmail.com (J.M.B.); ewelinadziedzic82@gmail.com (E.A.D.); 2 Centre of Postgraduate Medical Education (CMKP), 01-813 Warsaw, Poland

**Keywords:** coronary artery disease, acute coronary syndrome, myocardial infarction, depression, trace elements, zinc, selenium, magnesium, copper, oxidative stress, inflammation

## Abstract

Coronary artery disease (CAD), including acute coronary syndromes, frequently co-occurs with depression and is associated with adverse outcomes. Trace elements may influence shared biological pathways, including oxidative stress, inflammation, and neurovascular signaling. This study evaluated the association between trace element status and depressive symptoms in CAD. A systematic review was conducted in accordance with PRISMA 2020 guidelines and prospectively registered in PROSPERO (CRD420251231129). PubMed, Scopus, and the Cochrane Library were searched from inception to 2 December 2025. Studies assessing trace element concentrations in adults with CAD and depressive symptoms were eligible. Due to limited direct evidence, partially aligned and indirect studies were also included. Data were synthesized narratively. Of 699 records, four studies were included. No studies fulfilled Tier 1 criteria. The available evidence consisted of partially aligned (Tier 2) and indirect (Tier 3) studies. Lower zinc and magnesium levels and higher copper concentrations were suggested to be associated, based exclusively on Tier 2–3, low-certainty, predominantly indirect evidence. Interventional studies reported modest improvements following zinc or combined magnesium and zinc supplementation, although not in CAD-specific populations. Evidence directly addressing trace elements and depression in CAD is extremely limited and largely indirect. Current data do not support causal inference or clinical recommendations. Findings should be considered exploratory and hypothesis-generating.

## 1. Introduction

Coronary artery disease (CAD), including acute coronary syndromes, remains a leading cause of morbidity and mortality worldwide [1,2]. In contemporary clinical terminology, CAD encompasses a spectrum of atherosclerotic conditions, including chronic coronary syndromes and acute coronary syndromes, reflecting heterogeneous disease phenotypes [2,3]. In this review, the term CAD is used pragmatically to capture this spectrum, although it does not allow for precise differentiation between specific clinical entities. This limitation reflects the available literature, which rarely distinguishes between phenotypic subtypes in studies evaluating trace element status and depressive symptoms.

Depressive symptoms frequently co-occur in patients with CAD and are associated with adverse clinical outcomes, including reduced treatment adherence and increased cardiovascular risk [4,5,6]. Depression is particularly common following acute coronary events, with reported prevalence rates ranging from approximately 20% to 40%, and has been consistently associated with increased risk of recurrent cardiovascular events and mortality [7,8]. This comorbidity is increasingly conceptualized within a bidirectional heart–brain axis involving shared biological pathways [3,9,10,11].

Among the mechanisms proposed to underlie this interaction, chronic low-grade inflammation, oxidative stress, endothelial dysfunction, and neurovascular signaling disturbances are most consistently implicated [12,13,14,15,16]. At the molecular level, these processes involve activation of redox-sensitive transcription factors such as nuclear factor kappa B (NF-κB) and impaired antioxidant responses mediated by nuclear factor erythroid 2-related factor 2 (Nrf2), contributing to sustained inflammatory signaling, endothelial injury, and altered neuronal plasticity [6,12,15,17,18,19,20].

Emerging evidence suggests that macrophage dysfunction may contribute to persistent inflammation and cardiovascular remodeling, processes that may be influenced by trace elements involved in redox regulation [21,22,23,24].

Trace elements are essential micronutrients involved in redox homeostasis, immune regulation, and neuronal function. Elements such as zinc, magnesium, copper, and selenium modulate oxidative stress responses, inflammatory signaling pathways, and synaptic plasticity [25,26,27,28,29,30]. Through their effects on redox balance, mitochondrial function, and immune cell activity, disturbances in trace element homeostasis may influence biological processes relevant to both cardiovascular disease and depression [5,6,15,26,29,31]. Trace elements have been implicated in both cardiovascular and neuropsychiatric processes; however, their role in the combined CAD–depression phenotype remains poorly defined [26,31].

Despite these mechanistic insights, evidence directly linking trace element status with depressive symptoms in patients with coronary artery disease remains limited, fragmented, and largely indirect. Therefore, this systematic review aims to evaluate the available evidence on this association, with particular emphasis on the scarcity and indirect nature of the currently available data.

## 2. Materials and Methods

### 2.1. Protocol and Registration

The methodology of this systematic review adheres to the Preferred Reporting Items for Systematic Reviews and Meta-Analyses (PRISMA 2020) statement [32]. To ensure transparency and prevent duplication, the study protocol was prospectively registered with the International Prospective Register of Systematic Reviews (PROSPERO; Registration ID: CRD420251231129).

**Figure 1:**
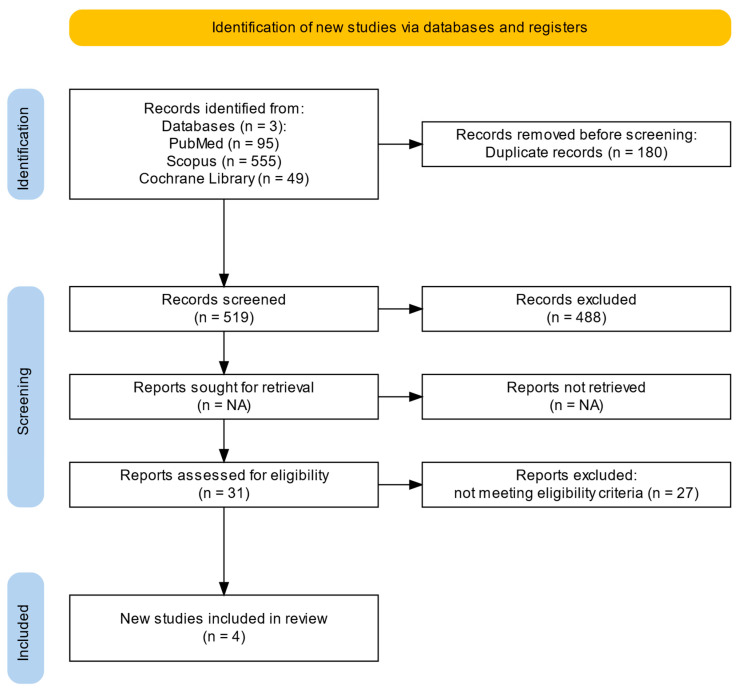
PRISMA flow diagram of the study selection process [33].

Given the limited number of studies directly addressing patients with co-occurring coronary artery disease and depression, a clarification regarding the inclusion of partially eligible studies providing indirect mechanistic evidence was documented prior to full-text synthesis. No other deviations from the registered protocol were made. The study selection process is presented in Figure 1.

### 2.2. Eligibility Criteria

For an appropriate selection of evidence, specific eligibility criteria were established based on the PICO (Population, Intervention/Exposure, Comparator, Outcome) framework. Studies were eligible for inclusion if they focused on adult populations diagnosed with coronary artery disease (CAD), including unstable angina (UA) and/or myocardial infarction (MI), with coexisting clinically diagnosed depression or depressive symptoms assessed using validated diagnostic criteria or standardized instruments (e.g., DSM/ICD-based diagnosis or validated rating scales, with reported scoring thresholds when applicable). Although the eligibility criteria covered a broad range of trace elements and metals, the included literature ultimately focused on essential trace elements, particularly zinc, magnesium, and copper. Studies specifically addressing toxic metals, including lead, cadmium, and mercury, were not identified as eligible and were therefore not included in the synthesis.

The review further included records assessing trace element concentration levels (zinc, selenium, magnesium, copper, iron, manganese, chromium, cobalt, cadmium, lead, mercury, arsenic, nickel, vanadium, iodine, boron, molybdenum, fluoride) measured in serum, plasma, erythrocytes/whole blood, hair, or urine. Both observational study designs (cohort, case–control, and cross-sectional) and interventional studies (randomized and non-randomized supplementation trials) were eligible. Interventional studies were prespecified for narrative synthesis due to anticipated heterogeneity in supplementation regimens (dose, formulation, and duration), study populations, and depression outcome measures, which precluded meaningful quantitative pooling.

The search was restricted to original research publications in English, without geographic or institutional setting limitations. Secondary literature, including systematic and narrative reviews, meta-analyses, consensus statements, book chapters, case reports, conference abstracts, editorials, and letters to the editor, was excluded.

Given the very limited number of studies directly addressing patients with co-occurring coronary artery disease and depression, studies partially fulfilling the eligibility criteria were also considered if they provided indirect but mechanistically relevant evidence regarding the association between trace elements and depressive symptoms. Such studies included investigations conducted in cardiometabolic populations without formally confirmed comorbid depression or studies assessing depressive outcomes in non-CAD cohorts. These records were included solely to support biological plausibility and mechanistic interpretation and were clearly identified as providing indirect evidence; they were not used to draw direct clinical conclusions regarding the combined CAD–depression phenotype.

### 2.3. Information Sources

A comprehensive literature search was conducted in PubMed, Scopus, and the Cochrane Library from database inception to 2 December 2025. The final search was performed on 2 December 2025, and all records retrieved up to that date were considered for screening.

To enhance completeness, backward citation searching of the reference lists of included studies and relevant reviews was additionally performed to identify potentially eligible records not captured through electronic database searches.

### 2.4. Search Strategy

To ensure the highest possible sensitivity and retrieval of relevant studies, a comprehensive search was conducted across PubMed, Scopus, and the Cochrane Library using database-specific search syntaxes tailored to the indexing architecture of each platform. In PubMed, searches combined MeSH terms with free-text keywords in titles and abstracts, while Scopus searches used TITLE-ABS-KEY fields and chemical identifiers due to the absence of a controlled vocabulary. In the Cochrane Library, exploded MeSH descriptors were combined with title, abstract, and keyword searches. Database-specific Boolean strategies were applied to integrate trace elements, coronary artery disease, and depression concepts, ensuring reproducibility of literature retrieval, with full electronic search strategies detailed in Table 1. Searches were limited to human studies published in English, and completeness was enhanced through backward citation searching of included studies and relevant reviews.

### 2.5. Study Selection

All records identified through database searching were imported into the screening platform, and duplicates were removed prior to the screening process.

The study selection process was performed independently by two reviewers (D.G. and Z.W.) using the Rayyan web-based platform, based on predefined eligibility criteria. Any disagreements regarding study inclusion were resolved through discussion or, when necessary, by consultation with a third independent reviewer (J.B.), who acted as an adjudicator.

Inter-rater agreement for title and abstract screening was assessed retrospectively using Cohen’s kappa coefficient, based on independently recorded reviewer decisions prior to consensus. Cohen’s κ was 0.50, indicating moderate agreement, with a high observed agreement of 99.3%. The difference between κ and observed agreement likely reflects the strong predominance of exclusion decisions across screened records, which is known to influence kappa values in imbalanced datasets.

### 2.6. Data Extraction

Data extraction was performed systematically using a standardized digital form to ensure accuracy and inter-reviewer consistency. The extraction form was pilot-tested on a subset of included studies prior to full data extraction to ensure clarity and consistency of data collection.

Two reviewers independently (J.B. and M.Ś.) extracted the following information from each eligible study: author and year of publication, country, study design, population characteristics (sample size, age, and clinical phenotype), depression assessment tools, trace elements assessed and corresponding biological matrices (including distinction between direct biochemical measurements and indirect exposure proxies), key findings including direction of association and reported effect sizes, and statistical adjustment strategies.

Trace element assessment methods were categorized as either direct biochemical measurements, such as serum or plasma concentrations, or indirect proxy measures derived from dietary assessment tools such as food frequency questionnaires or the Dietary Inflammatory Index.

To ensure transparency and consistency with the predefined data synthesis framework, each study was additionally classified according to its level of evidence (Tier 1–3) based on alignment with the primary PICO criteria, and the degree of PICO alignment (direct, partial, or indirect) was recorded.

Discrepancies in extracted data were resolved through discussion; unresolved discrepancies were adjudicated by a third author (Z.W.).

### 2.7. Risk of Bias

All included studies underwent a structured risk-of-bias assessment to support interpretation of the findings and to evaluate methodological quality. Risk-of-bias assessments were not used as exclusion criteria but were considered during qualitative synthesis and discussion of the results.

For observational studies, the Newcastle–Ottawa Scale (NOS) was applied. For cross-sectional studies, an adapted version of the NOS was used in accordance with previously published methodological recommendations [34,35]. For randomized controlled trials, the Risk of Bias 2 (RoB 2) tool was applied [36,37]. Risk of bias was assessed independently at the study level by two of three reviewers (D.G., J.B., or Z.W.), with disagreements resolved through discussion. Inter-rater agreement was evaluated using Cohen’s kappa coefficient, indicating substantial agreement between reviewers.

Risk-of-bias assessments were used to support interpretation of findings, while the primary weighting of evidence was based on its directness to the CAD–depression phenotype, as defined by the evidence tiering framework.

### 2.8. Data Synthesis Plan

Data synthesis was conducted qualitatively. Given the small number of eligible studies and substantial methodological and clinical heterogeneity, meta-analysis was not considered appropriate. A formal quantitative assessment of statistical heterogeneity (e.g., I^2^) was not performed because meta-analysis requires at least two studies with comparable populations, exposures, and outcome measures. In the present review, such comparability was not met.

Heterogeneity was assessed qualitatively across predefined domains, including population characteristics (coronary artery disease vs. non-coronary populations), exposure assessment (direct biochemical measurement vs. indirect dietary proxies), depression assessment methods (BDI, BDI-II, PHQ-9), and study design (observational vs. interventional). Substantial clinical and methodological heterogeneity across these domains precluded meaningful quantitative pooling of results.

To improve interpretability and reduce the risk of scope drift, included studies were categorized according to predefined levels of evidence based on their alignment with the primary PICO framework:

- Tier 1 (direct evidence): studies including patients with confirmed coronary artery disease and coexisting depression or depressive symptoms, with direct biochemical assessment of trace element concentrations;
- Tier 2 (partially direct evidence): studies conducted in coronary artery disease or cardiometabolic populations with incomplete alignment to the primary PICO criteria, including those in which depressive outcomes were secondary or where interventions involved combined supplementation;
- Tier 3 (indirect evidence): studies conducted in non-coronary populations or using indirect exposure measures, such as dietary-based indices, providing supportive but non-specific evidence.

As no studies fulfilled Tier 1 criteria, no direct conclusions could be drawn. Tier 2 and Tier 3 evidence were used solely to provide contextual and hypothesis-generating insights. Given the limited number of eligible studies and the predominance of indirect evidence, the review also incorporates elements of a scoping approach in order to contextualize biological plausibility and evidence gaps.

Narrative synthesis followed predefined grouping criteria, including population characteristics (coronary artery disease versus non-coronary populations), type of exposure assessment (biochemical measurement versus indirect proxy), method of depression assessment, and study design (observational versus interventional). Differences between studies were interpreted in relation to these domains.

Heterogeneity was observed across multiple domains, including the trace elements assessed, biological matrices used, methods of depression assessment (BDI, BDI-II, PHQ-9), and study populations. Accordingly, findings were synthesized narratively to allow comparison across heterogeneous designs and to support cautious interpretation of the available evidence regarding the association between trace element status and depression or depressive symptoms in the context of coronary artery disease.

It should be noted that the synthesis was restricted to essential trace elements, as no eligible studies investigating toxic metals were identified.

## 3. Results

### 3.1. Study Selection

The initial search identified 699 records (95 from PubMed, 555 from Scopus, and 49 from the Cochrane Library). After duplicate removal, 519 records were screened. Following title and abstract screening, 488 records were excluded for not meeting the eligibility criteria. Full-text assessment was performed for 31 articles, of which 27 were excluded. Ultimately, four studies were included in the systematic review.

None of the included studies fully met the predefined eligibility criteria. All four studies were therefore classified as providing either partially aligned (Tier 2) or indirect (Tier 3) evidence and were included to support an exploratory, hypothesis-generating synthesis.

### 3.2. Study Characteristics

The main characteristics of the included studies are presented in Table 2. Studies were categorized according to predefined evidence tiers (Tier 1–3), as described in the Data Synthesis Plan. No studies fulfilled Tier 1 criteria. Two studies were classified as Tier 2 (partially aligned evidence), and two as Tier 3 (indirect evidence).

The included studies were published between 2020 and 2024 and conducted in the Middle East and South Asia. Two studies had cross-sectional designs, and two were randomized controlled trials with intervention durations of 12 weeks. Sample sizes ranged from 60 to 5984 participants.

Depressive symptoms were assessed using validated instruments across studies, most commonly the Beck Depression Inventory (BDI or BDI-II), while one study used the Patient Health Questionnaire-9 (PHQ-9) [38]. Trace element exposure was assessed either through direct biochemical measurements, including serum zinc, magnesium, and copper, or indirectly using dietary-based indices. Study populations varied and included patients with coronary or cardiometabolic conditions as well as community-based cohorts.

**Table 2:** Characteristics of included studies, evidence tier classification, and relevance to the primary review question.

Author (Year), Country	Study Design	Population Characteristics (Number, Clinical Phenotype, Age)	Depression Assessment Tool	Trace Elements, Biological Matrix, and Assessment Method	Key Findings (Direction and Effect Size)	Evidence Tier	PICO Alignment	Statistical Adjustments
Mousa et al. (2022), Iraq [39]	Cross-sectional	*N* = 120 (UA/ATS) + 58 controls Age not specified	BDI-II (Moderate/Severe)	Zn, Mg, Cu serum (biochemical measurement)	Significant ↓ in Zn and Mg and ↑ in Cu in depressed UA patients (*p* < 0.001).	T2	Partial	Primary group comparisons adjusted for age, sex, and smoking status after log-transformation of trace element concentrations; additional multivariable regression and PLS path analyses incorporated cardiometabolic and inflammatory covariates.
Hamedifard et al. (2020), Iran * [40]	RCT (12-week)	*N* = 60 (CAD + T2DM); age: 45–95	BDI score	Mg + Zn serum (biochemical measurement)	Co-supplementation (250 mg MgO + ZnSO4) significantly ↓ BDI scores vs. placebo (−4.5 vs. −0.5, *p* < 0.04).	T2	Partial	ANCOVA adjusted for baseline BDI, BAI, and metabolic parameters
Yosaee et al. (2020), Iran [41]	RCT (12-week)	*N* = 140 (overweight/obese adults with mild–moderate depression **); mean age: 38.35 ± 6.70 years	BDI-II score (inclusion criterion: ≥10)	Zn serum (biochemical measurement)	Zinc supplementation (30 mg/day) was associated with a significant ↓ in BDI-II scores (*p* < 0.05).	T3	Indirect	ANCOVA adjusted for baseline BDI-II score and baseline serum zinc and vitamin D levels
Mahajan et al. (2024), India [38]	Cross-sectional (APCAPS cohort)	*N* = 5984 (Urbanizing community) Mean age: not reported	PHQ-9 (depression defined ≥5)	Fe, Mg, Se, Zn (Dietary Inflammatory Index ***; FFQ-based proxy measure)	Higher pro-inflammatory diet (DII) linked to ↑ odds of cardio-metabolic + mental comorbid.	T3	Indirect	Age, sex, education, tobacco use, alcohol intake, physical activity, BMI, and total energy intake (methods: Statistical analysis)

↑, increased; ↓, decreased;* Depressive symptoms in Hamedifard et al. were assessed as a secondary outcome rather than an inclusion criterion. ** Yosaee et al. included participants without diagnosed coronary artery disease. *** DII: Dietary Inflammatory Index (dietary proxy measure). PLS—partial least squares; APCAPS—Andhra Pradesh Children and Parents’ Study [42]. Trace element concentrations and units are reported as presented in the original studies due to heterogeneity in measurement methods and reporting standards. Biochemical measurements refer to direct assessment in biological matrices—serum or plasma—whereas dietary-based indices (e.g., Dietary Inflammatory Index) represent indirect proxy measures derived from food frequency questionnaires (FFQ).

### 3.3. Findings—Observational Studies

The observational evidence comprised two cross-sectional studies classified as Tier 2 and Tier 3, respectively.

The study by Mousa et al. [39] (Tier 2) was a cross-sectional study with clinical comparison groups that assessed associations between trace element concentrations and depressive symptom severity in a population of 178 individuals, including patients with atherosclerosis with and without unstable angina and healthy controls (Table 2). Depressive symptoms were evaluated using the Beck Depression Inventory-II (BDI-II). Serum concentrations of zinc, copper, calcium, and magnesium were measured using spectrophotometric methods.

A graded increase in depressive symptom severity was observed across clinical subgroups, with higher BDI-II scores in patients with atherosclerosis compared with controls, and the highest scores in those with unstable angina (*p* < 0.001). Lower serum zinc and magnesium concentrations and higher copper levels were associated with greater depressive symptom severity. In multivariable analyses, severe depressive symptoms were additionally associated with increased levels of interleukin-6 (IL-6), mu-opioid receptor (MOR), and markers of lipid peroxidation. These findings should be interpreted cautiously, as they derive from partially aligned (Tier 2) evidence and do not represent direct CAD–depression associations. This study provided partially aligned evidence, as the coronary phenotype was not strictly defined and depression was assessed as a severity gradient rather than a clearly defined comorbid condition.

The study by Mahajan et al. [38] (Tier 3), in turn, was a large cross-sectional analysis conducted in 5984 adults from a community-based cohort (Table 2). Depressive symptoms were assessed using the Patient Health Questionnaire-9 (PHQ-9). Trace element exposure was not measured directly but was estimated using the Dietary Inflammatory Index (DII) derived from a food frequency questionnaire, reflecting the inflammatory potential of the diet.

Higher DII scores were associated with increased odds of cardiometabolic and mental health multimorbidity after adjustment for demographic and lifestyle factors (*p* < 0.05). This study provided indirect evidence, as it was conducted in a non-coronary population and relied on dietary proxy measures rather than direct biochemical assessment of trace elements.

### 3.4. Findings—Interventional Studies

The interventional evidence comprised two randomized controlled trials classified as Tier 2 and Tier 3, respectively.

The study by Hamedifard et al. [40] (Tier 2) was a 12-week randomized, double-blind, placebo-controlled trial conducted in 60 patients with type 2 diabetes mellitus and coronary heart disease (Table 2). Participants received either combined supplementation with magnesium oxide (250 mg/day) and zinc sulfate (150 mg/day) or placebo. Depressive symptoms were assessed as a secondary outcome using the Beck Depression Inventory (BDI), and serum magnesium and zinc concentrations were measured at baseline and after the intervention.

In adjusted analyses, combined magnesium and zinc supplementation was associated with a statistically significant reduction in BDI scores compared with placebo (β = −3.61; *p* < 0.001), with a mean decrease of −4.5 points in the intervention group versus −0.5 points in the placebo group (*p* < 0.04). Improvements in anxiety and sleep quality were also reported. This study provided partially aligned evidence, as depression was not a defining inclusion criterion and the intervention involved combined supplementation, limiting attribution to individual trace elements.

The study by Yosaee et al. [41] (Tier 3) was a 12-week randomized, double-blind, placebo-controlled trial with a factorial design conducted in 140 overweight or obese adults with depressive symptoms but without diagnosed coronary artery disease (Table 2). Participants received zinc supplementation (30 mg/day), vitamin D (2000 IU/day), a combination of both, or placebo. Depressive symptom severity was assessed using the Beck Depression Inventory-II (BDI-II), and serum zinc concentrations were measured.

Zinc supplementation was associated with a statistically significant reduction in depressive symptom scores compared with placebo (*p* < 0.05), with improvements observed across intervention groups. This study provided indirect evidence, as it was conducted in a non-coronary population and therefore does not directly inform the coronary artery disease–depression phenotype.

To improve interpretability of the limited and heterogeneous evidence base, a structured evidence map summarizing the direction, strength, and level of evidence for individual trace elements is presented in Table 3.

### 3.5. Risk of Bias

All included studies underwent a structured risk-of-bias assessment in accordance with the predefined methodology. The Risk of Bias 2 (RoB 2) tool was applied to the randomized controlled trials by Hamedifard et al. [40] and Yosaee et al. [41]. The Newcastle–Ottawa Scale (NOS) adapted for cross-sectional studies was applied to the studies by Mahajan et al. [38] and Mousa et al. [39].

In the Newcastle–Ottawa Scale, three domains were evaluated: selection, comparability, and outcome. According to the adopted scoring system, studies were classified as very good (9–10 points), good (7–8 points), satisfactory (5–6 points), or unsatisfactory (0–4 points). Mahajan et al. [38] received a ‘Good’ rating, whereas Mousa et al. [39] received a ‘Satisfactory’ rating.

For the RoB 2 tool, Hamedifard et al. [40] and Yosaee et al. [41] were assessed across the domains of randomization process, deviations from intended interventions, missing outcome data, measurement of outcomes, and selection of the reported results. Both studies were rated as having a low risk of bias across all assessed domains.

**Figure 2:**
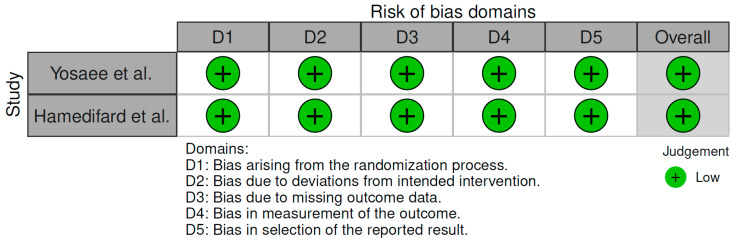
Results of individual study assessments done with RoB 2. Refs. [36,37,39,40,43].

The results of the risk-of-bias assessment are presented graphically in Table 4 (Newcastle–Ottawa Scale) and Figure 2 (RoB 2, visualized using the robvis tool).

## 4. Discussion

### 4.1. Principal Findings

The present review did not identify any studies fulfilling Tier 1 criteria, directly evaluating trace element status in patients with clearly defined coronary artery disease and depression or depressive symptoms using biochemical assessment. The available evidence consisted of two Tier 2 studies providing partially aligned data and two Tier 3 studies providing indirect evidence. Accordingly, the current literature does not directly address the primary review question and is limited to Tier 2–3, low to very low certainty, predominantly indirect evidence, allowing only cautious, hypothesis-generating interpretation.

Based exclusively on Tier 2–3, low-certainty and predominantly indirect evidence, alterations in zinc, magnesium, and copper concentrations should be interpreted as suggested but highly uncertain and non-phenotype-specific signals. In the observational study by Mousa et al. [39], lower circulating zinc and magnesium levels and higher copper concentrations were associated with greater depressive symptom burden in patients with atherosclerosis and unstable angina; however, the coronary phenotype was not strictly defined and depression was assessed as a severity gradient rather than a clearly defined comorbid condition. In the interventional study by Hamedifard et al. [40], combined magnesium and zinc supplementation was associated with modest reductions in depressive symptom scores, although depression was not a primary inclusion criterion and the use of combined supplementation limits attribution to individual elements.

The Tier 3 evidence provided additional indirect support from non-coronary populations and proxy exposure measures. The population-based study by Mahajan et al. [38] demonstrated an association between pro-inflammatory dietary patterns and cardiometabolic and mental health multimorbidity, although trace element exposure was assessed indirectly and individual micronutrient effects could not be isolated. Similarly, the randomized trial by Yosaee et al. [41] showed reductions in depressive symptom scores following zinc supplementation in overweight or obese individuals without coronary artery disease, limiting its applicability to the target phenotype.

Taken together, the available evidence suggests that zinc- and magnesium-related pathways may be relevant to depressive symptomatology in broader cardiometabolic contexts; however, this signal derives exclusively from Tier 2–3, low-certainty, indirect evidence and remains insufficiently specific to CAD populations. In the absence of Tier 1 studies, these observations should be interpreted as low-confidence, hypothesis-generating signals rather than phenotype-specific findings in patients with coronary artery disease and coexisting depression. Data for other trace elements remain sparse and largely indirect.

### 4.2. Biological Plausibility

Given the absence of Tier 1 evidence directly linking trace element status with depression or depressive symptoms in patients with coronary artery disease (CAD), the following mechanisms are presented to contextualize biological plausibility rather than to support direct clinical inference.

Zinc and copper serve as essential cofactors for antioxidant enzymes such as Cu/Zn superoxide dismutase (SOD), which catalyzes the dismutation of superoxide radicals and mitigates oxidative stress [44]. Dysregulation of these elements may impair antioxidant defense, leading to increased reactive oxygen species (ROS) production, reduced nitric oxide bioavailability, and activation of redox-sensitive inflammatory pathways, including NF-κB signaling [44,45,46,47]. Alterations in the copper-to-zinc ratio have been associated with systemic oxidative stress and inflammatory states in both cardiovascular and psychiatric populations [48,49,50].

Magnesium plays a central role in cellular energy metabolism and mitochondrial function by stabilizing MgATP^2−^ complexes and supporting enzymatic activity [51]. Magnesium deficiency has been linked to increased oxidative stress, endothelial dysfunction, and activation of pro-inflammatory pathways, including elevated IL-6 and TNF-α levels [52,53]. It may also contribute to dysregulation of the hypothalamic–pituitary–adrenal (HPA) axis, influencing neuroendocrine stress responses relevant to both depressive symptomatology and cardiometabolic dysfunction [29,54,55].

Disturbances in trace element homeostasis may further modulate inflammatory signaling through activation of the NLRP3 inflammasome, which has been implicated in both neuroinflammatory processes and atherosclerotic plaque instability [56]. Zinc deficiency may enhance ROS production and NF-κB activation, whereas magnesium deficiency may increase susceptibility to inflammasome activation [57,58,59].

In addition to these pathways, emerging evidence highlights the role of macrophage functional and metabolic reprogramming in cardiovascular pathology. Epigenetic regulation of macrophage efferocytosis has been shown to impair the clearance of apoptotic cells and promote persistent inflammation within atherosclerotic lesions [21,22]. Furthermore, activation of fatty acid synthesis pathways in macrophages contributes to pathogenic fibroblast expansion and fibrotic remodeling following myocardial infarction [23,24]. Macrophage function is closely dependent on intracellular redox balance, mitochondrial activity, and metabolic regulation—processes in which trace elements such as zinc and magnesium play essential regulatory roles [25,26,27,28,29].

Beyond systemic inflammation, trace elements may influence neurotransmission and synaptic plasticity. Zinc and magnesium modulate glutamatergic signaling, including N-methyl-D-aspartate receptor (NMDAR) activity, which plays a role in neuronal excitability and synaptic function [34,60,61]. Dysregulation of these pathways may contribute to impaired neuroplasticity and depressive symptomatology.

Dietary patterns influencing micronutrient intake may further contribute to systemic inflammation. Higher Dietary Inflammatory Index scores have been associated with both cardiovascular disease and depression [62,63], potentially through mechanisms involving TLR4/NF-κB signaling and alterations in trace element distribution [64,65,66].

**Figure 3:**
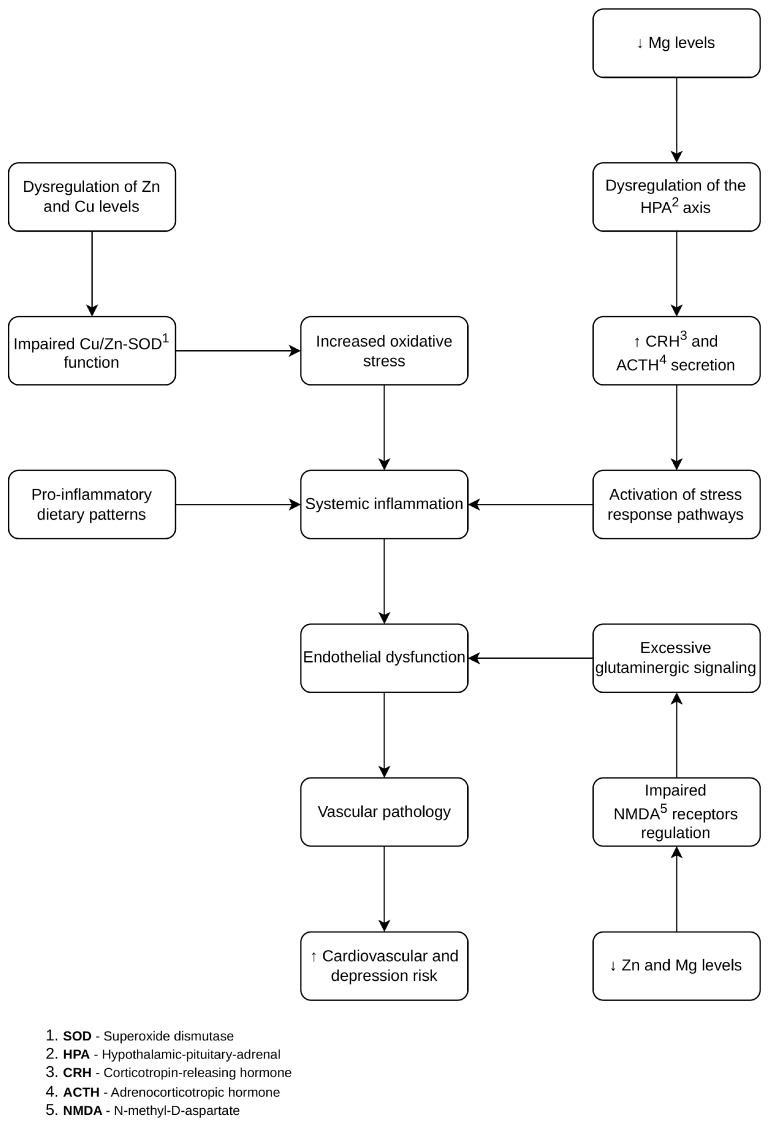
Proposed biological pathways linking trace element dysregulation with coronary artery disease and depressive symptoms. The figure represents a conceptual framework based on available evidence and is intended to illustrate potential mechanisms. It does not represent a causal model established by the included studies.

Taken together, these mechanisms support a biologically plausible link between trace element imbalance, vascular dysfunction, and depressive symptomatology. However, most of these pathways have been described in experimental or indirect clinical contexts and have not been directly demonstrated in patients with coexisting CAD and depression or depressive symptoms. Accordingly, these mechanisms should be interpreted as conceptual and hypothesis-generating. The proposed pathways are illustrated in Figure 3.

### 4.3. Methodological and Conceptual Barriers Limiting Available Evidence

The limited number of studies examining trace element status in patients with coronary artery disease (CAD) and coexisting depression or depressive symptoms reflects broader methodological and conceptual challenges inherent to this research field. Cardiovascular and psychiatric conditions are typically investigated within separate clinical and scientific frameworks, which restricts the availability of integrated datasets. Cardiovascular studies often do not include systematic assessment of depressive symptoms, whereas psychiatric research rarely incorporates detailed cardiovascular phenotyping. This separation limits the ability to evaluate shared biological mechanisms within the same patient populations.

Additional complexity arises from heterogeneity in exposure assessment. Trace element status is measured using a variety of biological matrices, including serum, plasma, whole blood, hair, or urine, and in some cases is inferred indirectly from dietary-based indices such as food frequency questionnaires or the Dietary Inflammatory Index. The absence of standardized protocols, reporting units, and analytical methods reduces comparability across studies and complicates interpretation.

Conceptual heterogeneity is also evident in the definition of clinical populations. The term coronary artery disease encompasses a spectrum of conditions, including chronic coronary syndromes and acute coronary syndromes, which represent heterogeneous atherosclerotic phenotypes. However, most studies do not provide sufficient phenotypic resolution to allow for meaningful subgroup analyses.

Finally, research in this area has predominantly focused on individual trace elements rather than integrated mineral profiles or interaction patterns, despite evidence that trace elements act within complex biological networks. These methodological and conceptual challenges contribute to the limited availability and comparability of evidence and should be considered when interpreting the findings.

### 4.4. Comparison with Related Evidence

To our knowledge, no systematic review or meta-analysis has specifically examined trace element status in patients with co-occurring coronary artery disease and depression or depressive symptoms. Consistent with the absence of Tier 1 evidence identified in the present review, available literature addressing trace elements in this context remains fragmented and is largely derived from studies focusing separately on either cardiovascular or psychiatric populations.

The studies included in this review primarily investigated zinc, magnesium, and copper, with iron and selenium assessed to a lesser extent. However, as these findings were derived exclusively from Tier 2 and Tier 3 evidence, they should not be interpreted as specific to the coronary artery disease–depression phenotype. For example, Mousa et al. [39] reported lower zinc and magnesium levels and higher copper concentrations in patients with atherosclerosis and depressive symptoms, while Hamedifard et al. [40] and Yosaee et al. [41] observed reductions in depressive symptom scores following zinc-containing supplementation in selected populations. These findings are consistent with broader literature suggesting a potential role of trace elements in mood regulation but remain non-specific to coronary artery disease.

Previous studies have reported associations between zinc deficiency and depressive symptoms [67], as well as between magnesium deficiency and both cardiovascular risk and depression-related outcomes [68,69]. Similarly, elevated copper levels have been linked to depressive disorders [49] and increased cardiovascular risk [70]. However, these studies were conducted in separate clinical contexts and do not directly address the combined CAD–depression phenotype.

Accordingly, the apparent overlap between trace element–related mechanisms in cardiovascular and psychiatric conditions should be interpreted as contextual rather than confirmatory. In the absence of studies directly evaluating both conditions within the same population, current evidence supports biological plausibility but does not establish a phenotype-specific association.

### 4.5. Clinical Implications

In the absence of Tier 1 evidence directly evaluating trace element status in patients with coronary artery disease and coexisting depression or depressive symptoms, the clinical relevance of the available findings remains uncertain. The current evidence base consists exclusively of partially aligned (Tier 2) and indirect (Tier 3) studies, which do not allow for phenotype-specific inference.

Accordingly, there is insufficient evidence to support routine assessment of trace element status or the use of targeted micronutrient supplementation in patients with coronary artery disease and depression or depressive symptoms. The available data do not justify the implementation of micronutrient-based therapeutic strategies in clinical practice.

At present, the potential role of trace elements should be considered hypothesis-generating. Future research should focus on well-designed prospective studies and randomized controlled trials conducted in clearly characterized coronary artery disease populations with coexisting depression or depressive symptoms, incorporating direct biochemical assessment and standardized clinical endpoints. Until such data are available, any clinical application should be approached with caution.

Importantly, the recently published 2025 ESC Clinical Consensus Statement on mental health and cardiovascular disease does not address the role of trace elements in this context, which is consistent with the current lack of clinically actionable evidence and highlights the gap between mechanistic plausibility and guideline-supported practice [71].

### 4.6. Limitations

This systematic review has several important limitations that should be considered when interpreting the findings.

First, no studies fulfilled Tier 1 criteria, indicating a complete absence of direct evidence evaluating trace element status in patients with coronary artery disease and coexisting depression or depressive symptoms using biochemical measurements. The available evidence was limited to partially aligned (Tier 2) and indirect (Tier 3) studies, substantially restricting the strength and specificity of the conclusions. Importantly, despite the use of structured risk-of-bias tools, the overall methodological quality of the included studies remains limited, particularly due to small sample sizes, heterogeneity, and the predominance of indirect evidence.

Second, the included studies were heterogeneous in terms of population characteristics, exposure assessment, outcome definitions, and study design. This heterogeneity, together with the small number of eligible studies, precluded formal quantitative synthesis, limited the applicability of standard heterogeneity metrics such as I^2^, and reduced the generalizability of the findings to the CAD–depression phenotype.

Third, differences in exposure assessment represent an important limitation. Trace element status was evaluated either through direct biochemical measurements or indirectly using dietary-based proxies, which reflect distinct constructs and are not directly comparable.

Fourth, although risk-of-bias assessment was performed using validated tools (Newcastle–Ottawa Scale and RoB 2), the included studies varied in methodological quality and were predominantly cross-sectional or indirectly aligned with the predefined PICO framework, further limiting causal interpretation.

Fifth, the use of the term coronary artery disease reflects the terminology applied in the available literature; however, the included studies did not allow for consistent differentiation between specific clinical phenotypes, such as chronic coronary syndromes and acute coronary syndromes.

Sixth, although both essential trace elements and toxic metals may influence overlapping biological pathways, the present review is limited to essential trace elements, such as zinc, magnesium, and copper, as no eligible studies investigating toxic metals were identified.

Finally, the overall certainty of the evidence should be considered low to very low, primarily due to indirectness, heterogeneity, and the limited number of eligible studies.

Taken together, these limitations indicate that the current evidence base is limited, heterogeneous, and predominantly indirect, and does not allow for causal inference or clinically actionable conclusions.

### 4.7. Research Recommendations

The absence of Tier 1 studies directly evaluating trace element status in patients with coronary artery disease and coexisting depression or depressive symptoms highlights a substantial gap in the current literature and underscores the need for more integrated study designs.

Future research should prioritize prospective cohort studies specifically enrolling patients with clearly defined coronary artery disease and coexisting depression or depressive symptoms, using standardized diagnostic criteria or validated instruments. Longitudinal assessment of trace element status before and after major cardiovascular events may help distinguish pre-existing deficiencies from secondary alterations related to acute illness, inflammation, or pharmacotherapy.

Greater standardization in trace element assessment is essential, including consistent selection of biological matrices, harmonized reporting units, and transparent description of analytical methods. In parallel, detailed cardiovascular phenotyping and objective measures of disease severity should be incorporated to ensure precise characterization of the target population.

Future investigations should also move beyond single-element analyses and instead evaluate broader mineral profiles and interaction patterns, such as copper–zinc ratios or multi-element panels, to better reflect systemic redox and inflammatory regulation. Careful adjustment for key confounding factors—including diet, smoking, medication use (e.g., statins, antidepressants, and antidiabetic agents), renal function, and inflammatory markers—is necessary to improve causal inference.

Finally, adequately powered randomized controlled trials conducted in well-characterized coronary artery disease populations with coexisting depression or depressive symptoms are required to determine whether targeted modulation of trace element status has clinically meaningful effects on mood, cardiovascular outcomes, or both.

## 5. Conclusions

Despite growing interest in the role of trace elements in mental health, no studies were identified that directly evaluated the association between trace element status and depression or depressive symptoms in patients with clearly defined coronary artery disease using biochemical assessment (Tier 1 evidence). The available literature consisted exclusively of partially aligned (Tier 2) and indirect (Tier 3) studies.

Tier 2 evidence, which remains low-certainty and only partially aligned with the primary review question, suggested, based exclusively on Tier 2–3, low-certainty evidence, that alterations in zinc, magnesium, and copper status may be associated with depressive symptom severity in coronary or cardiometabolic contexts; however, these observations are exploratory and should not be interpreted as phenotype-specific to patients with coronary artery disease and depression or depressive symptoms. Tier 3 evidence provided additional indirect support from non-coronary populations and dietary proxy measures but does not allow for CAD-specific inference.

Accordingly, current evidence does not permit causal interpretation or support clinical recommendations regarding trace element assessment or supplementation in patients with coronary artery disease and coexisting depression or depressive symptoms. The principal contribution of this review is the identification of a substantial evidence gap in this field.

Future research should focus on well-designed prospective studies and randomized controlled trials in clearly characterized coronary artery disease populations with coexisting depression or depressive symptoms, using direct biochemical assessment of trace element status. Until such data are available, existing findings should be interpreted as exploratory and hypothesis-generating.

## Figures and Tables

**Table 1:** Complete search strategies for PubMed, Scopus, and the Cochrane Library, adapted to the indexing and search characteristics of each database.

**PubMed**		
(“Trace Elements”[Mesh] OR trace element*[tiab] OR “Micronutrients”[Mesh] OR micronutrient*[tiab] OR zinc[Mesh] OR zinc[tiab] OR selenium[Mesh] OR selenium[tiab] OR magnesium[Mesh] OR magnesium[tiab] OR copper[Mesh] OR copper[tiab] OR iron[Mesh] OR iron[tiab] OR manganese[Mesh] OR manganese[tiab] OR chromium[Mesh] OR chromium[tiab] OR cobalt[Mesh] OR cobalt[tiab] OR cadmium[Mesh] OR cadmium[tiab] OR mercury[Mesh] OR mercury[tiab] OR arsenic[Mesh] OR arsenic[tiab] OR nickel[Mesh] OR nickel[tiab] OR vanadium[Mesh] OR vanadium[tiab] OR iodine[Mesh] OR iodine[tiab] OR boron[Mesh] OR boron[tiab] OR molybdenum[Mesh] OR molybdenum[tiab] OR fluoride[Mesh] OR fluoride[tiab] OR lead [Mesh] OR lead [Substance Name]) AND (“Coronary Artery Disease”[Mesh] OR coronary artery disease[tiab] OR “Acute Coronary Syndrome”[Mesh] OR acute coronary syndrome[tiab] OR “Myocardial Infarction”[Mesh] OR myocardial infarction[tiab] OR STEMI[tiab] OR “ST Elevation Myocardial Infarction”[MeSH] OR NSTEMI[tiab] OR “Non-ST Elevation Myocardial Infarction”[MeSH] OR unstable angina[tiab] OR “Angina, Unstable”[Mesh] OR atherosclerosis[tiab] OR “Atherosclerosis”[Mesh] OR myocardial ischemia[tiab] OR “Myocardial Ischemia”[Mesh] OR CAD[tiab] OR CHD[tiab] OR chronic coronary syndrome[tiab]) AND (“Depression”[Mesh] OR depression[tiab] OR “Depressive Disorder”[Mesh] OR depressive disorder[tiab] OR “Mood Disorders”[Mesh] OR mood disorder*[tiab])		
**Scopus**		
(TITLE-ABS-KEY(“Trace Elements”) OR TITLE-ABS-KEY(“trace element*”) OR TITLE-ABS-KEY(micronutrient*) OR TITLE-ABS-KEY(zinc) OR TITLE-ABS-KEY(selenium) OR TITLE-ABS-KEY(magnesium) OR TITLE-ABS-KEY(copper) OR TITLE-ABS-KEY(iron) OR TITLE-ABS-KEY(manganese) OR TITLE-ABS-KEY(chromium) OR TITLE-ABS-KEY(cobalt) OR TITLE-ABS-KEY(cadmium) OR TITLE-ABS-KEY(mercury) OR TITLE-ABS-KEY(arsenic) OR TITLE-ABS-KEY(nickel) OR TITLE-ABS-KEY(vanadium) OR TITLE-ABS-KEY(iodine) OR TITLE-ABS-KEY(boron) OR TITLE-ABS-KEY(molybdenum) OR TITLE-ABS-KEY(fluoride) OR (CASREGNUMBER(7439-92-1) OR CHEMNAME(lead))) AND (TITLE-ABS-KEY(“Coronary Artery Disease”) OR TITLE-ABS-KEY(“Acute Coronary Syndrome”) OR TITLE-ABS-KEY(“Myocardial Infarction”) OR TITLE-ABS-KEY(“ST Elevation Myocardial Infarction”) OR TITLE-ABS-KEY(“Non-ST Elevation Myocardial Infarction”) OR TITLE-ABS-KEY(“Angina, Unstable”) OR TITLE-ABS-KEY(“Atherosclerosis”) OR TITLE-ABS-KEY(“Myocardial Ischemia”) OR TITLE-ABS-KEY(“coronary artery disease”) OR TITLE-ABS-KEY(“acute coronary syndrome”) OR TITLE-ABS-KEY(“myocardial infarction”) OR TITLE-ABS-KEY(STEMI) OR TITLE-ABS-KEY(NSTEMI) OR TITLE-ABS-KEY(“unstable angina”) OR TITLE-ABS-KEY(atherosclerosis) OR TITLE-ABS-KEY(“myocardial ischemia”) OR TITLE-ABS-KEY(CAD) OR TITLE-ABS-KEY(CHD) OR TITLE-ABS-KEY(“chronic coronary syndrome”) OR TITLE-ABS-KEY(“coronary disease”)) AND (TITLE-ABS-KEY(“Depression”) OR TITLE-ABS-KEY(“Depressive Disorder”) OR TITLE-ABS-KEY(“Mood Disorders”) OR TITLE-ABS-KEY(depression) OR TITLE-ABS-KEY(“depressive disorder”) OR TITLE-ABS-KEY(“mood disorder*”))		
**Cochrane Library**		
**Search ID**	**Search strategy**	**Results**
#1	MeSH descriptor: [Trace Elements] explode all trees	619
#2	(trace element*):ti,ab,kw	1350
#3	MeSH descriptor: [Micronutrients] explode all trees	7147
#4	(micronutrient*):ti,ab,kw	4232
#5	MeSH descriptor: [Zinc] explode all trees	2048
#6	(zinc):ti,ab,kw	7654
#7	MeSH descriptor: [Selenium] explode all trees	949
#8	(selenium):ti,ab,kw	2524
#9	MeSH descriptor: [Magnesium] explode all trees	1543
#10	(magnesium):ti,ab,kw	10,652
#11	MeSH descriptor: [Copper] explode all trees	621
#12	(copper):ti,ab,kw	2633
#13	MeSH descriptor: [Iron] explode all trees	3267
#14	(iron):ti,ab,kw	13,828
#15	MeSH descriptor: [Manganese] explode all trees	92
#16	(manganese):ti,ab,kw	456
#17	MeSH descriptor: [Chromium] explode all trees	433
#18	(chromium):ti,ab,kw	1534
#19	MeSH descriptor: [Cobalt] explode all trees	337
#20	(cobalt):ti,ab,kw	1186
#21	MeSH descriptor: [Cadmium] explode all trees	78
#22	(cadmium):ti,ab,kw	204
#23	MeSH descriptor: [Mercury] explode all trees	96
#24	(mercury):ti,ab,kw	1503
#25	MeSH descriptor: [Arsenic] explode all trees	92
#26	(arsenic):ti,ab,kw	514
#27	MeSH descriptor: [Nickel] explode all trees	427
#28	(nickel):ti,ab,kw	950
#29	MeSH descriptor: [Vanadium] explode all trees	16
#30	(vanadium):ti,ab,kw	71
#31	MeSH descriptor: [Iodine] explode all trees	1732
#32	(iodine):ti,ab,kw	6298
#33	MeSH descriptor: [Boron] explode all trees	31
#34	(boron):ti,ab,kw	289
#35	MeSH descriptor: [Molybdenum] explode all trees	35
#36	(molybdenum):ti,ab,kw	170
#37	MeSH descriptor: [Lead] explode all trees	191
#38	MeSH descriptor: [Fluorides] explode all trees	3449
#39	(fluoride):ti,ab,kw	6401
#40	MeSH descriptor: [Coronary Artery Disease] explode all trees	9686
#41	(coronary artery disease):ti,ab,kw	28,756
#42	MeSH descriptor: [Acute Coronary Syndrome] explode all trees	3166
#43	(acute coronary syndrome):ti,ab,kw	8940
#44	MeSH descriptor: [Myocardial Infarction] explode all trees	15,779
#45	(myocardial infarction):ti,ab,kw	39,051
#46	(STEMI):ti,ab,kw	4590
#47	MeSH descriptor: [ST Elevation Myocardial Infarction] explode all trees	1216
#48	(ST elevation myocardial infarction):ti,ab,kw	7965
#49	(NSTEMI):ti,ab,kw	911
#50	MeSH descriptor: [Non-ST Elevated Myocardial Infarction] explode all trees	208
#51	(Non-ST elevation myocardial infarction):ti,ab,kw	2156
#52	(unstable angina):ti,ab,kw	5066
#53	MeSH descriptor: [Angina, Unstable] explode all trees	1430
#54	(atherosclerosis):ti,ab,kw	12,154
#55	MeSH descriptor: [Atherosclerosis] explode all trees	3917
#56	("myocardial ischemia"):ti,ab,kw	7572
#57	MeSH descriptor: [Myocardial Ischemia] explode all trees	39,120
#58	(CAD):ti,ab,kw	7066
#59	(CHD):ti,ab,kw	4070
#60	(chronic coronary syndrome):ti,ab,kw	1276
#61	(coronary disease):ti,ab,kw	43,954
#62	MeSH descriptor: [Depression] explode all trees	19,843
#63	(depression):ti,ab,kw	117,711
#64	MeSH descriptor: [Depressive Disorder] explode all trees	17,302
#65	(depressive disorder):ti,ab,kw	27,686
#66	MeSH descriptor: [Mood Disorders] explode all trees	21,048
#67	(mood disorder*):ti,ab,kw	14,246
#68	#1 OR #2 OR #3 OR #4 OR #5 OR #6 OR #7 OR #8 OR #9 OR #10 OR #11 OR #12 OR #13 OR #14 OR #15 OR #16 OR #17 OR #18 OR #19 OR #20 OR #21 OR #22 OR #23 OR #24 OR #25 OR #26 OR #27 OR #28 OR #29 OR #30 OR #31 OR #32 OR #33 OR #34 OR #35 OR #36 OR #37 OR #38 OR #39	58,471
#69	#40 OR #41 OR #42 OR #43 OR #44 OR #45 OR #46 OR #47 OR #48 OR #49 OR #50 OR #51 OR #52 OR #53 OR #54 OR #55 OR #56 OR #57 OR #58 OR #59 OR #60 OR #61	90,459
#70	#62 OR #63 OR #64 OR #65 OR #66 OR #67	128,535
#71	#68 AND #69 AND #70	49

**Table 3:** Evidence map of trace elements and depressive symptoms across included studies, stratified by evidence tier and PICO alignment.

Element	No. of Studies	Evidence Tiers	Direction of Association/Effect	CAD-Specific Evidence	Confidence Level	Main Limitation
Zinc	3	T2–T3	Suggested association with lower depressive symptoms	No	Low to very low (indirect, non-CAD-specific)	Predominantly indirect populations; supplementation studies outside CAD
Magnesium	2	T2	Suggested association with lower depressive symptoms	Partial	Low (partially indirect; combined interventions)	Combined supplementation; depressive outcomes often secondary
Copper	1	T2	Suggested association with greater depressive symptom severity	Limited	Low (single, indirect study)	Cross-sectional design; indirect CAD–depression linkage
Iron/Selenium (via DII)	1	T3	Indirect association (dietary pattern)	No	Very low (proxy exposure; indirect)	Indirect exposure (dietary proxy); no biochemical measurement

T2—partially direct evidence; T3—indirect evidence. No studies fulfilled Tier 1 criteria. Direction reflects within-study trends and should be interpreted cautiously given the limited, heterogeneous and predominantly indirect evidence base. All signals should be considered exploratory and hypothesis-generating rather than phenotype-specific.

**Table 4:** Risk of bias assessment of observational studies using an adapted Newcastle–Ottawa Scale for cross-sectional studies [34,35].

Ref.	Selection				Comparability	Outcome		Total
	Representativeness of the Sample	Sample Size	Non- Respondents	Ascertainment of Exposure	Based on Design and Analysis	Assessment of Outcome	Statistical Test	
Mousa et al. (2022) [39]				++	++	+	+	6 points (Satisfactory studies)
Mahajan et al. (2024) [38]	+			++	++	+	+	7 points (Good studies)

The Newcastle–Ottawa Scale (NOS) adapted for cross-sectional studies was used to assess methodological quality across three domains: selection, comparability, and outcome. The adapted version allows a maximum score of 10 points. Higher scores indicate lower risk of bias. Symbols: “++” indicates that the criterion was fully met, “+” indicates partial fulfillment, and blank indicates that the criterion was not met. Quality categories (e.g., “good”, “satisfactory”) were assigned for descriptive purposes only and should be interpreted with caution given the limited number of studies and methodological heterogeneity.

## Data Availability

No new data were created or analyzed in this study.

## Abbreviations

The following abbreviations are used in this manuscript:

ANCOVA	Analysis of covariance
APCAPS	Andhra Pradesh Children and Parents’ Study
ATS	Atherosclerosis
BAI	Beck Anxiety Inventory
BDI	Beck Depression Inventory
BDI-II	Beck Depression Inventory–II
BMI	Body Mass Index
CAD	Coronary Artery Disease
Cu	Copper
DII	Dietary Inflammatory Index
DSM	Diagnostic and Statistical Manual of Mental Disorders
ESC	European Society of Cardiology
Fe	Iron
FFQ	Food Frequency Questionnaire
HPA	Hypothalamic–Pituitary–Adrenal
ICD	International Classification of Diseases
IL-6	Interleukin-6
Mg	Magnesium
MgATP^2−^	Magnesium–Adenosine Triphosphate Complex
MgO	Magnesium Oxide
MI	Myocardial Infarction
MOR	Mu-Opioid Receptor
NF-κB	Nuclear Factor Kappa B
NLRP3	NOD-like receptor family pyrin domain containing 3
NMDAR	N-Methyl-D-Aspartate Receptor
NOS	Newcastle–Ottawa Scale
NSTEMI	Non-ST-Elevation Myocardial Infarction
Nrf2	Nuclear Factor Erythroid 2-Related Factor 2
PHQ-9	Patient Health Questionnaire-9
PICO	Population, Intervention/Exposure, Comparator, Outcome
PLS	Partial Least Squares
PRISMA	Preferred Reporting Items for Systematic Reviews and Meta-Analyses
PROSPERO	International Prospective Register of Systematic Reviews
RCT	Randomized Controlled Trial
RoB 2	Risk of Bias 2
ROS	Reactive Oxygen Species
SOD	Superoxide Dismutase
STEMI	ST-Elevation Myocardial Infarction
T2DM	Type 2 Diabetes Mellitus
TLR4	Toll-Like Receptor 4
TNF-α	Tumor Necrosis Factor alpha
UA	Unstable Angina
Zn	Zinc
ZnSO_4_	Zinc Sulfate
